# Historical overview of key issues in food safety.

**DOI:** 10.3201/eid0304.970410

**Published:** 1997

**Authors:** E M Foster

**Affiliations:** Food Research Institute, University of Wisconsin, Madison 53706, USA.

## Abstract

Foodborne transmission of pathogenic and toxigenic microorganisms has been a recognized hazard for decades. Even half a century ago we knew about the dangers of botulism from underprocessed canned foods; staphylococcal poisoning from unrefrigerated cream-filled pastries, sliced ham, meat, and poultry salads; and salmonellosis from infected animal products. Despite new protective measures, changes in preservation techniques and failure to follow recognized procedures have created new dangers. Moreover, we now recognize new organisms that can cause foodborne illness--Listeria monocytogenes, Escherichia coli O157:H7, Campylobacter jejuni, Vibrio parahaemolyticus, Yersinia enterocolitica, and others. Controlling these organisms will require widespread education and possibly new regulatory initiatives.


### Historical Overview of Key Issues in Food Safety

**E. M. Foster**
Madison, Wisconsin, USA

Foodborne transmission of pathogenic and toxigenic microorganisms has been a recognized hazard for decades. Even half a century ago we knew about the dangers of botulism from underprocessed canned foods; staphylococcal poisoning from unrefrigerated cream-filled pastries, sliced ham, meat, and poultry salads; and salmonellosis from infected animal products. Despite new protective measures, changes in preservation techniques and failure to follow recognized procedures have created new dangers. Moreover, we now recognize new organisms that can cause foodborne illness—*Listeria monocytogenes, Escherichia coli* O157:H7*, Campylobacter jejuni, Vibrio parahaemolyticus, Yersinia enterocolitica*, and others. Controlling these organisms will require widespread education and possibly new regulatory initiatives.

When I was growing up on my parents' farm in East Texas, we never thought about food poisoning or unsafe food. The only foods we bought were sugar, salt, flour, and oatmeal; everything else we produced and preserved on the farm. My mother spent all summer canning fruits and vegetables for winter. We had no refrigeration; we cured our own meat and drank raw milk. But I never heard of botulism, staph poisoning, or salmonellosis or perfringens poisoning until I studied bacteriology in college. Only then did I wonder how we survived with no refrigeration in a hot climate. Finally, the answer came to me. We just did not give the bacteria time enough to develop so they could hurt us. Leftovers from breakfast—hot biscuits, eggs, ham, bacon or sausage, oatmeal, coffee or milk—went right out to the chickens. Lunch leftovers—biscuits, cornbread, vegetables, or fried chicken—were saved for a cold supper 4 or 5 hours later. Any food left went to the pigs. The bacteria had only a maximum of 3 or 4 hours to grow, and that usually is not enough. I survived and went on to study food microbiology, which included what was known then about food poisoning. The guru of food poisoning in those days was professor Gail M. Dack at the University of Chicago. Dr. Dack was a protégé of Professor E.O. Jordan, who in 1917 published a 107-page book entitled Food Poisoning. Dr. Dack took over the book and published his first version of Food Poisoning in 1943. In 1949 and 1956, subsequent editions appeared in which certain truisms became apparent.

Botulism was considered a problem of canners, both home and commercial. Thus, adequate heat processing would seem to solve the problem. Perhaps it did for the canner, but now we know that heating will not eliminate all botulism. Many foods, including salmon eggs, smoked fish, garlic in oil, vacuum packaged lotus roots, and baked potatoes, can support growth and botulinum toxin formation if the storage temperature is suitable. Similarly, we thought staphylococcal poisoning was limited to cream-filled pastries and cured ham. In recent years, outbreaks of staphylococcal poisoning have been traced to cheese, whipped butter, ham salad, fermented sausages, and canned corned beef. We now know how to prevent staphylococcal poisoning, but not all food handlers understand and fully comply with the appropriate control measures.

Salmonellosis was once thought a problem with meat from infected animals. Now we know that a variety of food products can serve as vehicles of this disease. As early as World War II, we found that dried eggs from the United States could transmit this disease to our British allies. Thousands of cases of human salmonellosis in the United States and other industrialized countries

Address for correspondence: E. M. Foster, Professor Emeritus, Food Research Institute, University of Wisconsin, Madison, WI 53706 USA; fax: 608-263-1114.

have been transmitted by ice cream, chocolate, potato salad, cheddar cheese, raw milk, black pepper, pâté, aspic, ham, pasteurized milk, and drinking water.

*Clostridium perfringens,* known since the 1940s, causes a problem only when there is gross temperature abuse of cooked food. *Clostridium botulinum, Staphylococcus aureus, C. perfringens,* and the salmonellae were well known in Dr. Dack's day, although the food vehicles might have changed. Not so well known were many of the organisms that preoccupy us today. For example, we used to think of *Escherichia coli* as merely an indicator organism that suggested insanitary handling. Now we know forms of *E. coli* can kill. Thirty years ago, *Listeria monocytogenes, Campylobacter jejuni, Aeromonas hydrophila, Plesiomonas shigelloides, Vibrio parahaemolyticus,* and *Yersinia enterocolitica* were not known; now these are well-established foodborne pathogens that we must control.

Although not part of a historical overview, other key issues deserve attention during this meeting. For example, we once thought that fresh, uncracked eggs were essentially sterile and safe to eat. We did not recognize the ability of *Salmonella* Enteritidis to invade the laying hen and thereby the yolk of an egg. An outbreak of *S.* Enteritidis at a Chicago hotel taught us not to rely on the safety of eggs merely because the shell was intact. *S.* Enteritidis in shell eggs is still a serious health problem and a growing concern to egg and poultry producers.

Of equal, if not greater, concern is *Salmonella* Typhimurium strain DT 104. Widely distributed in cattle herds of England, Scotland, and Wales, this organism is resistant to several antibiotics, including ampicillin, chloramphenicol, streptomycin, sulfonamides, and tetracycline. Between 1990 and 1995, the number of *S.* Typhimurium DT 104 isolated from humans in Britain increased from 259 to 3,837 per year—a 15-fold increase. Moreover, the percentage of drug-resistant isolates increased from 39% in 1990 to 97% in 1995. *S.* Typhimurium DT 104 has been isolated in the United States from sheep, pigs, horses, goats, emus, cats, dogs, elk, mice, coyotes, ground squirrels, raccoons, chipmunks, and birds. American egg and poultry producers are concerned about its entry into U.S. poultry flocks. *S.* Typhimurium DT 104 infection in humans has been associated with the consumption of chicken, sausage, and meat paste as well as with the handling of sick animals. More than one-third of the patients have required hospitalization, and 3% have died; these figures are very unusual for ordinary Salmonella infections and indicate serious problems ahead.

